# Beyond treatment volume: when pelvic radiotherapy may still matter in high-risk localised prostate cancer

**DOI:** 10.3389/fonc.2026.1831135

**Published:** 2026-05-15

**Authors:** Iván Henríquez, Oscar Abuchaibe, Barbara Malave-Chacon, Meritxell Arenas

**Affiliations:** 1 Radiation Oncology Department, Institut de Recerca Biomèdica Catalunya Sud (IRB-CS), Hospital Universitari Sant Joan de Reus, Tarragona, Spain; 2 Radiation Oncology Department, FOSCAL Clinic, Bucaramanga, Colombia; 3 Radiation Oncology Department, Hospital Viamed, Tarragona, Spain

**Keywords:** androgen receptor pathway inhibitors, cN1 disease, genomics, high-risk prostate cancer, metastasis-free survival, precision medicine, PSMA PET, whole-pelvic radiotherapy

## 1 Introduction

The management of high-risk localised prostate cancer has evolved substantially over the past two decades, driven by advances in radiotherapy (RT), androgen deprivation therapy (ADT), and, more recently, systemic intensification with androgen receptor pathway inhibitors (ARPIs). Elective pelvic radiotherapy—whole-pelvic radiotherapy (WPRT)—has therefore remained a recurrent point of contention, oscillating between periods of widespread adoption and concerns about toxicity or limited incremental benefit. Reducing the debate to a binary choice (pelvis versus no pelvis) overlooks the biological, anatomical, and systemic complexity of high-risk disease. The clinically relevant question is not whether pelvic RT retains validity per se, but rather which patients—given the staging approach, systemic backbone, and tumour biology—are most likely to derive a net clinical benefit in terms of metastasis-free survival (MFS) and overall survival (OS) that justifies its use.

## 2 Subsections relevant to the subject

### 2.1 Historical foundations: RT and ADT as the turning point

Landmark randomised trials established that the greatest therapeutic gains in locally advanced and high-risk prostate cancer were achieved through systemic intensification with ADT added to RT. EORTC 22863 demonstrated substantial improvements in local control, progression-free survival, and OS with the addition of long-term ADT to RT, and RTOG 92–02 showed that prolonging adjuvant ADT after RT improved biochemical and clinical outcomes, particularly in high-grade disease (1, 2). While these trials were not designed to isolate the impact of pelvic field size, they delivered a consistent message: the dominant driver of benefit was systemic rather than purely geometric. This is reinforced by the MARCAP individual patient data meta-analysis, which showed that adding ADT to definitive RT and prolonging adjuvant ADT improves MFS, whereas intensification limited to neoadjuvant therapy does not confer a clear benefit (3).

### 2.2 Pelvic RT: heterogeneous evidence and why it diverges

In contrast to the ADT signal, the evidence base for WPRT has been heterogeneous. GETUG-01 did not demonstrate a global benefit in event-free survival or OS for elective pelvic irradiation compared with prostate-only RT (4). RTOG 94–13 highlighted that pelvic field size cannot be interpreted in isolation and may interact with ADT sequencing and duration, with a complex balance between tumour control and toxicity (5). In the modern era, POP-RT reported improvements in biochemical failure–free survival, disease-free survival, and distant metastasis–free survival with WPRT in carefully selected patients with a high probability of nodal involvement treated with intensity-modulated radiotherapy (IMRT) and prolonged hormonal therapy (6). POP-RT mandated a Roach-estimated pelvic nodal risk ≥20%, and a contemporaneous commentary noted a median nodal-risk estimate of approximately 38%, effectively enriching for patients with substantial occult nodal risk—an important contrast with broader favourable-risk populations, in which lower nodal event rates may attenuate any WPRT signal (7).

In this context, the results reported from NRG/RTOG 0924 (8) did not show an OS benefit for WPRT compared with prostate-only RT in patients with unfavourable intermediate-risk or favourable high-risk disease, demonstrating only a modest reduction in biochemical failure, no difference in metastasis rates, and higher rates of grade 2 gastrointestinal toxicity. These findings reinforce that WPRT should not be universally applied, and should redirect the debate towards patient selection (true nodal risk, cN1 disease) and integration with systemic intensification (9). While the higher incidence of grade 2 gastrointestinal toxicity reported in RTOG 0924 underscores the potential morbidity of pelvic volumes, the consistent use of advanced delivery techniques such as IMRT or volumetric modulated arc therapy (VMAT)—as employed in the POP-RT trial—remains essential to ensure that oncologic benefit effectively outweighs toxicity in carefully selected patients.

Notably, the settings in which pelvic RT appears most biologically plausible are those enriched for regional nodal disease—either clinically evident (cN1) or at high risk for occult nodal involvement—mirroring the POP-RT selection strategy and the hypothesis-generating cN1/pelvic-RT signals reported in ENZARAD. This supports a selection-based framework in which pelvic RT is considered preferentially when “true nodal risk” is high and systemic intensification is optimised. Contemporary pooled individual patient data analyses such as SANDSTORM further support a clinically meaningful interaction between irradiated volume and ADT sequencing for MFS and metastatic outcomes (9). Taken together, these data argue against universal WPRT while supporting a selective role in patients with a high likelihood of occult nodal disease, particularly when delivered with contemporary techniques to preserve the therapeutic ratio.

Although not performed in the intact-prostate setting, randomised evidence in regional recurrence supports the principle that elective pelvic nodal treatment can add benefit in higher-risk scenarios. In SPPORT, adding short-term ADT and pelvic nodal radiotherapy to prostate bed salvage RT improved freedom from progression compared with prostate bed RT alone, with acceptable late toxicity (10). Similarly, PEACE V–STORM reported improved metastasis-free survival with elective nodal radiotherapy compared with metastasis-directed therapy for PET-detected pelvic nodal oligorecurrence, both combined with 6 months of ADT (11). These contexts differ from definitive management, but they reinforce the biological plausibility of pelvic nodal coverage when regional disease burden is demonstrable.

### 2.3 Locoregional intensification beyond pelvic field size

Broadening the lens beyond pelvic field size alone also addresses a key limitation of many pelvic RT debates: WPRT is only one component of locoregional intensification. ASCENDE-RT demonstrated that escalating dose to the primary with a low-dose-rate (LDR) brachytherapy boost substantially improves biochemical control compared with a dose-escalated external beam boost in unfavourable-risk disease, albeit with higher genitourinary toxicity (12). PIVOTALboost is evaluating a factorial strategy of pelvic nodal treatment and intraprostatic boosting, explicitly testing both ‘“where” (field size) and “how much” (dose escalation) within a single programme (13). PACE-NODES is testing whether ultrahypofractionated prostate stereotactic body radiotherapy (SBRT) with pelvic nodal SBRT (both delivered in five fractions) improves biochemical/clinical failure–free outcomes compared with prostate-only SBRT in high-risk localised disease treated with long-term ADT (14). Finally, PEARLS extends the anatomic question by testing extended-field RT (including para-aortic nodes) in patients with prostate-specific membrane antigen (PSMA)-avid pelvic and/or para-aortic nodal disease at presentation, a population enriched for “true nodal risk” by molecular imaging (15). Importantly, these programmes also highlight that the marginal value of any locoregional intensification is conditional on baseline systemic control. When occult metastatic burden is the dominant driver, escalating local or nodal dose may primarily improve intermediate endpoints rather than MFS or OS. Conversely, in patients enriched for true nodal risk—particularly with PSMA PET—field selection (pelvic vs extended-field) becomes a testable hypothesis regarding eradication of regionally confined micrometastatic disease, and therefore a plausible partner to systemic intensification. In this framework, pelvic RT is best viewed as one element within a biologically informed treatment package (imaging-defined risk, locoregional intensification, and modern systemic backbone), rather than a standalone “yes/no” decision. These studies collectively underscore that the key clinical problem is not simply pelvic volume, but the optimal integration of locoregional intensification strategies with systemic therapy and patient selection (**Table 1**).

**Table 1:** Selected contemporary and ongoing trials informing locoregional intensification in high-risk prostate cancer.

Trial	Population/setting	RT strategy	Systemic backbone	Primary endpoint	Key take-home
POP-RT	High-risk; high nodal risk selection	WPRT vs PORT; IMRT	ADT (long-term)	BFFS	WPRT benefit in selected high nodal-risk
RTOG 94-13	High nodal-risk (LNI ≥15%)	WPRT vs PORT; sequencing	ADT (short-term; timing varied)	PFS	Field size interacts with ADT sequencing
ASCENDE-RT	Unfavourable-risk (incl. high-risk)	Pelvic RT + primary boost (LDR vs EBRT)	ADT	bPFS	Primary boost improves biochemical control
PIVOTALboost	Unfavourable intermediate/high-risk	Pelvis vs prostate ± focal boost	ADT (per protocol)	PFS/toxicity	Factorial test of nodes and boost
PEARLS	PSMA-avid pelvic/para-aortic nodes	Extended-field RT (para-aortic) vs standard	ADT ± AR-targeted therapy/docetaxel	PFS/MFS	Tests extended field in PSMA-enriched nodal disease
STAMPEDE (M0)	High-risk non-metastatic	RT per SOC (not field-randomised)	ADT + abiraterone ± enzalutamide	MFS	Systemic intensification improves MFS/OS
ENZARAD	High-risk localised/LA (incl. cN1)	RT + ADT ± enzalutamide	ADT ± ARPI	MFS	No global MFS benefit; exploratory heterogeneity
PACE-NODES	High-risk localised; radical SBRT platform	Prostate SBRT vs prostate + pelvic nodal SBRT (5 fractions)	Long-term ADT (≥12 months)	B/clinical FFS	Tests pelvic nodal SBRT in a 5-fraction platform
SPPORT (RTOG 0534)*	Post-prostatectomy salvage (PSA recurrence; N0/Nx)	PBRT ± short-term ADT ± pelvic nodal RT	ADT (4–6 months) (arms 2–3)	Freedom from progression	Incremental benefit when adding pelvic nodes with ADT
PEACE V–STORM*	PET-detected pelvic nodal oligorecurrence after local therapy	MDT vs ENRT (pelvis) + nodal boost	ADT (6 months) (both arms)	MFS	ENRT improved MFS vs MDT

*Supportive evidence outside the definitive setting (indirect).

ADT, androgen deprivation therapy; ARPI, androgen receptor pathway inhibitor; B/clinical FFS, biochemical/clinical failure–free survival; bPFS, biochemical progression-free survival; BFFS, biochemical failure–free survival; EBRT, external beam radiotherapy; ENRT, elective nodal radiotherapy; IMRT, intensity-modulated radiotherapy; LDR, low-dose-rate; MDT, metastasis-directed therapy; MFS, metastasis-free survival; PBRT, prostate bed radiotherapy; PLNRT, pelvic lymph node radiotherapy; PORT, prostate-only radiotherapy; PFS, progression-free survival; RT, radiotherapy; SBRT, stereotactic body radiotherapy; SOC, standard of care; WPRT, whole-pelvic radiotherapy; PSMA, prostate-specific membrane antigen. ENZARAD results are currently available in abstract form only.

### 2.4 Systemic intensification: lessons from STAMPEDE and ENZARAD

Second-generation ARPIs have redefined systemic management in high-risk non-metastatic disease. STAMPEDE showed that adding abiraterone (with or without enzalutamide) to standard ADT improves MFS and OS in high-risk M0 patients, many treated with RT, establishing a modern systemic benchmark (16). By contrast, ENZARAD (reported to date in abstract form) did not demonstrate a global improvement in MFS or OS with enzalutamide added to RT and standard ADT. However, exploratory analyses suggest heterogeneity, including potential signals in cN1 disease and in those treated with pelvic RT (17). These findings are hypothesis-generating and suggest that the incremental value of ARPI intensification may depend on the locoregional context and the underlying burden of regional disease, rather than indiscriminate escalation for all high-risk patients. Ongoing trials, such as DASL-HiCaP (NCT04136353), will further clarify whether better-tolerated AR antagonists (e.g., darolutamide) can improve outcomes when integrated with definitive RT in very high-risk settings.

### 2.5 Patient selection: molecular imaging and tumour biology

A central limitation of older pelvic-RT trials is imperfect nodal staging. PSMA PET has transformed the detection of subclinical nodal involvement, redefining “nodal risk” by moving beyond size-based CT/MRI criteria to a biologically informed denominator. Selection is also likely to improve as PSMA PET technology evolves. A recent prospective head-to-head comparison suggested higher patient-level positivity and lesion detection with 24-hour 64Cu-SAR-bisPSMA compared with standard 68Ga-PSMA-11 in a biochemical recurrence cohort (18). Although conducted outside primary staging, such findings illustrate how advances in tracer performance could further refine enrichment for “true nodal risk”. It is plausible that some heterogeneity across WPRT trials reflects misclassification of true N1 disease as N0. Beyond anatomy, tumour biology is increasingly relevant. Genomic classifier platforms and, more recently, somatic profiling with next-generation sequencing (NGS) may identify tumours with a higher propensity for regional versus systemic dissemination. Integrating molecular imaging, systemic intensification, and biological stratification could enable a precision approach to WPRT—treating those most likely to benefit while avoiding unnecessary toxicity (19).

## 3 Discussion

Overall, the available evidence suggests that therapeutic progress in high-risk prostate cancer has been driven—and will likely continue to be driven—by systemic intensification, first with prolonged ADT and now with ARPIs. Within this evolving landscape, WPRT should neither be abandoned nor applied indiscriminately. Instead, its use should be framed as one component of locoregional intensification whose benefit is most plausible when three conditions align: (i) a high probability of occult nodal disease (e.g., cN1 or high “true nodal risk”), (ii) delivery with modern techniques to preserve the therapeutic ratio, and (iii) integration with an effective systemic backbone.

Pragmatic implications (hypothesis-informed):

- Consider WPRT preferentially in patients with cN1 disease or a high probability of occult nodal involvement, particularly when supported by PSMA PET staging.
- Optimise the therapeutic ratio with IMRT/VMAT, robust image guidance, and strict bowel/bladder constraints.
- Recognise that locoregional escalation extends beyond pelvic volume: primary boosting strategies (e.g., brachytherapy boost) and, in selected PSMA-avid nodal disease cases, extended-field approaches are active areas of investigation.
- Treat WPRT as an integrated strategy with systemic intensification; ongoing trials will clarify the best ARPI backbones and sequencing.

Limitations include heterogeneity in trial populations, endpoints, RT techniques, and systemic backbones; limited availability of PSMA-guided randomised data; and the preliminary nature of evidence reported only in abstract form for some contemporary ARPI-RT trials. Despite these limitations, the weight of evidence supports a precision-medicine framework: the goal is not simply to irradiate larger volumes, but to select the right patient and integrate staging, systemic therapy, and tumour biology to maximise net clinical benefit while minimising toxicity.
